# The mixed phylogenetic origin of northern pike (*Esox lucius* Linnaeus 1758) populations in the Middle Danubian drainage

**DOI:** 10.1186/s40850-022-00129-6

**Published:** 2022-05-30

**Authors:** Péter Takács, Bálint Bánó, István Czeglédi, Tibor Erős, Árpád Ferincz, Blanka Gál, Bernadett Bánó-Kern, Balázs Kovács, András Attila Nagy, Krisztián Nyeste, Vera Lente, Bálint Preiszner, Sándor Sipos, Ádám Staszny, Zoltán Vitál, András Weiperth, Eszter Csoma

**Affiliations:** 1 Balaton Limnological Research Institute, Klebelsberg Kuno str. 3, Tihany, 8237 Hungary; 2 Department of Molecular Ecology, Institute of Aquaculture and Environmental Safety, Hungarian University of Agriculture and Life Sciences, Páter Károly str. 1, Gödöllő, 2103 Hungary; 3 Department of Freshwater Fish Ecology, Institute of Aquaculture and Environmental Safety, Hungarian University of Agriculture and Life Sciences, Páter Károly str. 1, Gödöllő, 2103 Hungary; 4 “MilvusGroup” Birdland Nature Protection Association, Crinului nr 22, 540343 Tîrgu Mureș, Romania; 5 Evolutionary Ecology Group, Hungarian Department of Biology and Ecology, Faculty of Biology and Geology, University Babeş-Bolyai, Strada Clinicilor 5–7, 400006 Cluj-Napoca-Napoca, Romania; 6 Department of Hydrobiology, Faculty of Science and Technology, University of Debrecen, Egyetem sqr. 1, Debrecen, 4032 Hungary; 7 Department of Biology and Ecology, Faculty of Sciences, University of Novi Sad, 21102 Novi Sad, Serbia; 8 Research Center for Fisheries and Aquaculture, Institute of Aquaculture and Environmental Safety, Hungarian University of Agricultural and Life Sciences, Anna-liget u. 35, Szarvas, 5540 Hungary; 9 Department of Medical Microbiology, Faculty of Medicine, University of Debrecen, Nagyerdei krt. 98, Debrecen, 4032 Hungary

**Keywords:** Cytochrome B, D-loop, Secondary contact, Hybridisation, Carpathian basin, Anthropogenic effect

## Abstract

**Background:**

Pikes, members of genus *Esox*, are widespread freshwater predators of the northern hemisphere, and important sport fish also. From the Carpathian basin only one species, the northern pike (*E. lucius*) is noted. At the same time the pike stocks living in this area show high level of phenotypic variance (e.g. various body pattern) and its growth varies highly both among and within populations. These features usually explained by the environmental diversity of the area. Whereas we think that genetic reasons -e.g. the appearance of other/new pike species in the area- may also be responsible for these observed features. Since as no detailed information have been published from the pike populations of this area, so far; we conducted phylogenetic and morphological assay on 88 pike specimens, collected from 49 Middle Danubian sampling sites.

**Results:**

Our phylogenetic surveys showed that the northern pike appear in the study area solely, but all the three of its major lineages (Northern, Circumpolar, Southern) were indicated. Only six specimens represent the Northern lineage, collected from the western part of the study area. The Circumpolar and Southern lineages were common in the Carpathian basin, but the Southern lineage showed higher levels of haplotype diversity than the Circumpolar clade. Which indicates that only the Southern lineage is native in the area, while the other two groups could have appeared in the Middle Danubian system either spontaneously or by human introduction. Moreover, the different clades appeared in the same populations, suggesting the opportunity of inter-lineage hybridisation. From the studied morphologicalal features, the number of scales on the lateral line and the head length showed significant differences among the lineages. At the same time the body pattern of the studied individuals seems to be rather influenced by the ontogenic changes than phylogeny.

**Conclusions:**

The high phenotypic variability of Middle Danubian northern pike populations may be due that all of its three major clades appeared and came in secondary contact in the area. In the within watershed spread of the non-native lineages the human stocking/transfer may play a considerable role.

**Supplementary Information:**

The online version contains supplementary material available at 10.1186/s40850-022-00129-6.

## Background

Pikes (Actinopterygii: Esocidae) belong to the most characteristic piscivorous fish of the northern hemisphere [1]. This ancient teleost family consists of only seven species, from which three can be found only in North America and three others appear in Eurasia [2]. The eponymous species of the Esociformes genus, the northern pike (*Esox lucius* Linnaeus, 1758) has a circumpolar distribution and wide ecological tolerance [3–5]. Northern pike is one of the most widespread predators of the European freshwaters; moreover, it is one of the most important game and commercial fish species in the area [6–8]. Hence, this species has both great ecological and economical importance. Additionally, in the recent years, pike has become a preferred model organism of ecological and evolutionary biological studies, therefore its scientific importance has been continuously increasing [9–11].

The northern pike was known for a long time as a widely distributed species in the northern hemisphere, and the only *Esox* species in Europe [12]. However, it is characterised by high phenotypic variability. For example, the number of scales on the lateral line and the number of vertebrae vary in a wide range, 105–148 and 56–65 respectively [3]. Studies using both molecular and morphological methods published in recent years have modified all the genetic, taxonomic and distribution features of this taxon greatly. The phylogenetic investigations of European *Esox* populations showed that pikes are among those taxa whose distribution and phylogeny had been considerably affected by paleoclimatic changes [13]. For example, the Atlantic areas of Europe, which were glaciated in the ice ages, host genetically much more uniform stocks than the South European drainages [14]. In the last comprehensive study, Skog et al. analysed samples from the whole distribution area of *E. lucius* [15]. Their results showed that this species separated into three major lineages (Circumpolar, Northern, and Southern) only in the geological near past, 0.18–0.26 Myr ago. The existence of the latter two lineages suggests that the postglacial colonization of Europe set off from two independent refugia, situated in Western Europe and in the Danube region. Moreover, it is proved that the circumpolar is the only northern pike lineage of Eastern Asia [16].

In the last decade, two new European pike species have been described based on the results of detailed phylogenetic works [17–19]. The *E. cisalpinus* Bianco & Delmastro, 2011 [17], and *E. aquitanicus* [19] supposedly survived the last glaciations in refugia situated in the Appennine peninsula and Southwestern France respectively. These two recently described species are characterised by specific body colour and pattern and lower scale number on the lateral line. Moreover the *E. aqutanicus*, has shorter preorbital region, and it has less vertebrae than the *E. lucius* [18, 20, 21].

Our study area, the Carpathian basin, is located in the middle Danubian drainage. This basin was never glaciated during the ice ages [22], therefore it might have served as an extra-Mediterranean refugium and source of recolonisation to northern European areas for many plant and animal taxa [23, 24]. As many suitable lowland aquatic habitats can be found in the Carpathian basin, the pike is one of the most frequent piscivorous fish species in this area, and additionally, it is one of the most popular game fish in Hungary [25, 26]. At the same time, Hungarian pike populations express a high degree of phenotypic plasticity [27]. Moreover, the growth and length–weight relationships of Hungarian pike populations vary greatly among and within sites [28, 29], which is generally explained by the highly variable environmental conditions of the area [30]. Furthermore, their colour and body patterns are highly diverse, and the number of scales on the lateral line shows high variability but often less than 120 [31], which makes these individuals similar to the newly described European *Esox* species. Parallelly, scarce genetic information is available regarding the pikes from the Carpathian basin. Only a few specimens were analysed in the work of Nicod et al. [14], and these samples showed high levels of phylogenetic variability. Moreover, some of the indicated haplotypes were highly different from the ones that originated from Western Europe.

Additionally to the confusing phylogenetic background, we experienced high phenotypic variability during regular sampling campaigns. Therefore, the presence of at least two or more phylogenetically and morphologicalally distinct pike clades were hypothesized in the study area. However, it is still not clarified if the assumed clades are identical to the lineages noted by Skog et al. [15] or if an unknown (relict?) pike taxon that could be found in the study area. Moreover, as the *E. cisalpinus* has already been noted from the River Danube [32], it cannot be ruled out that any of the newly described pike species may be present in our study area. Therefore, the present study aims to clarify the phylogenetic features of pike populations of the Carpathian basin and to reveal if the observed genetic differences may also manifest in phenotypic traits.

## Results

### Phylogenetic features

**Fig. 1:**
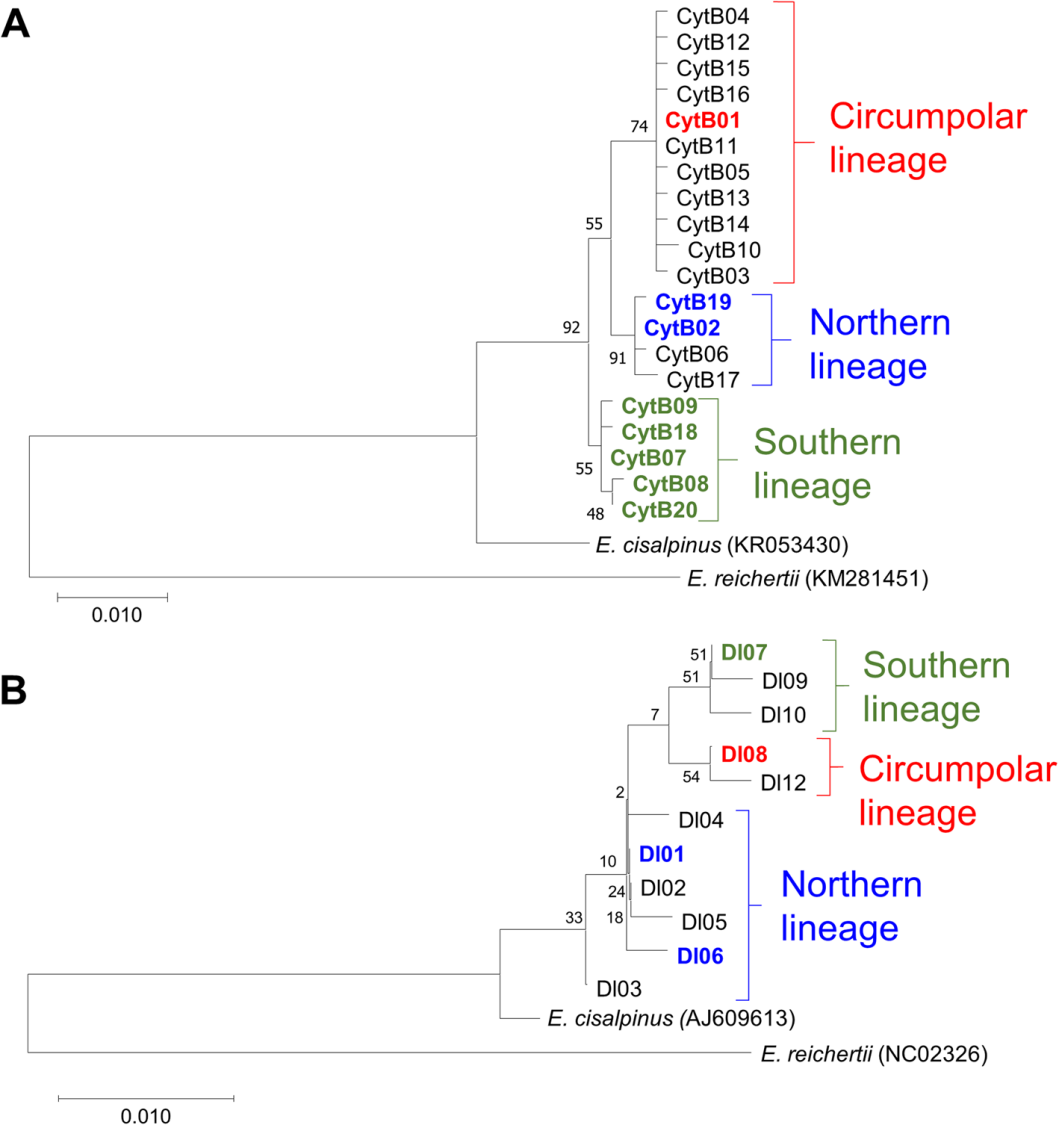
Evolutionary analysis of the indicated CytB (A) and D-loop (B) haplotypes by using the Maximum Likelihood method and Tamura-Nei model. The percentage of trees in which the associated taxa clustered together is shown next to the branches. The haplotypes appeared during our survey are highlighted by bold letter type. The lineage membership are shown by different colors (blue: Northern, red: Circumpolar, green: Southern). ML trees were built using *E. reichertii* and *E. cisalpinus* haplotypes as outgroup sequences, their Genbank accession numbers are indivcated in brackets. Colour figure online

**Fig. 2:**
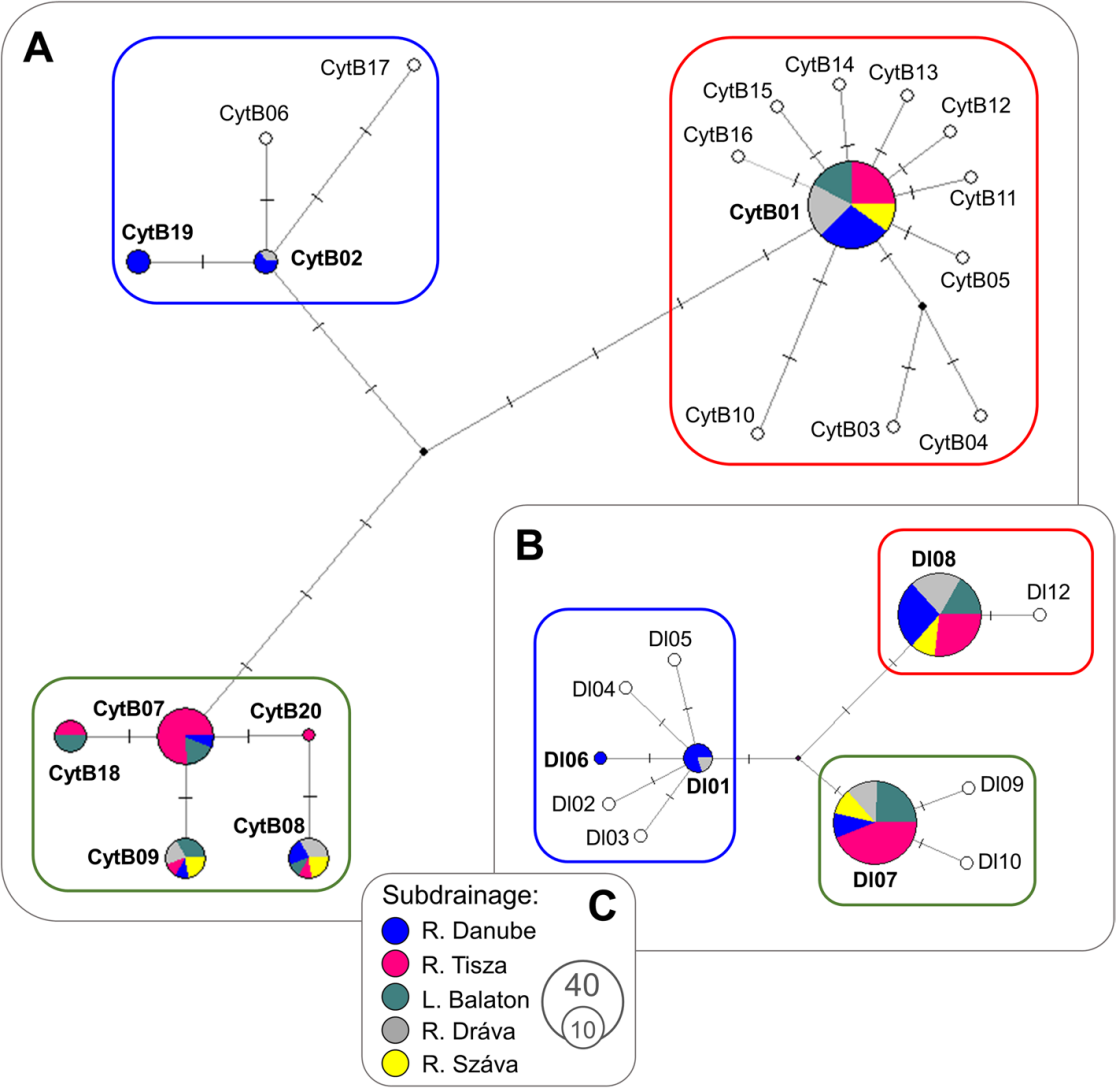
Median-Joining network of CytB (A) and D-loop (B) sequence data of the investigated 88 northern pike individuals. Line length refers to the genetic distances of haplotypes. Each vertical line is one mutation step. Small black circles represent missing or theoretical haplotypes. Haplotypes appeared from the study area marked by bold letter type. Haplotypes which were not indicated from the area are marked by white circles. The different lineages are emphasized with different coloured frames (blue: Northern, red: Circumpolar, green: Southern). The size and coloration of pie charts refer to the number of individuals carriing the certain haplotype and to their hydrographic origin (C). For more information see Table 1 and 2. Colour figure online

**Fig. 3:**
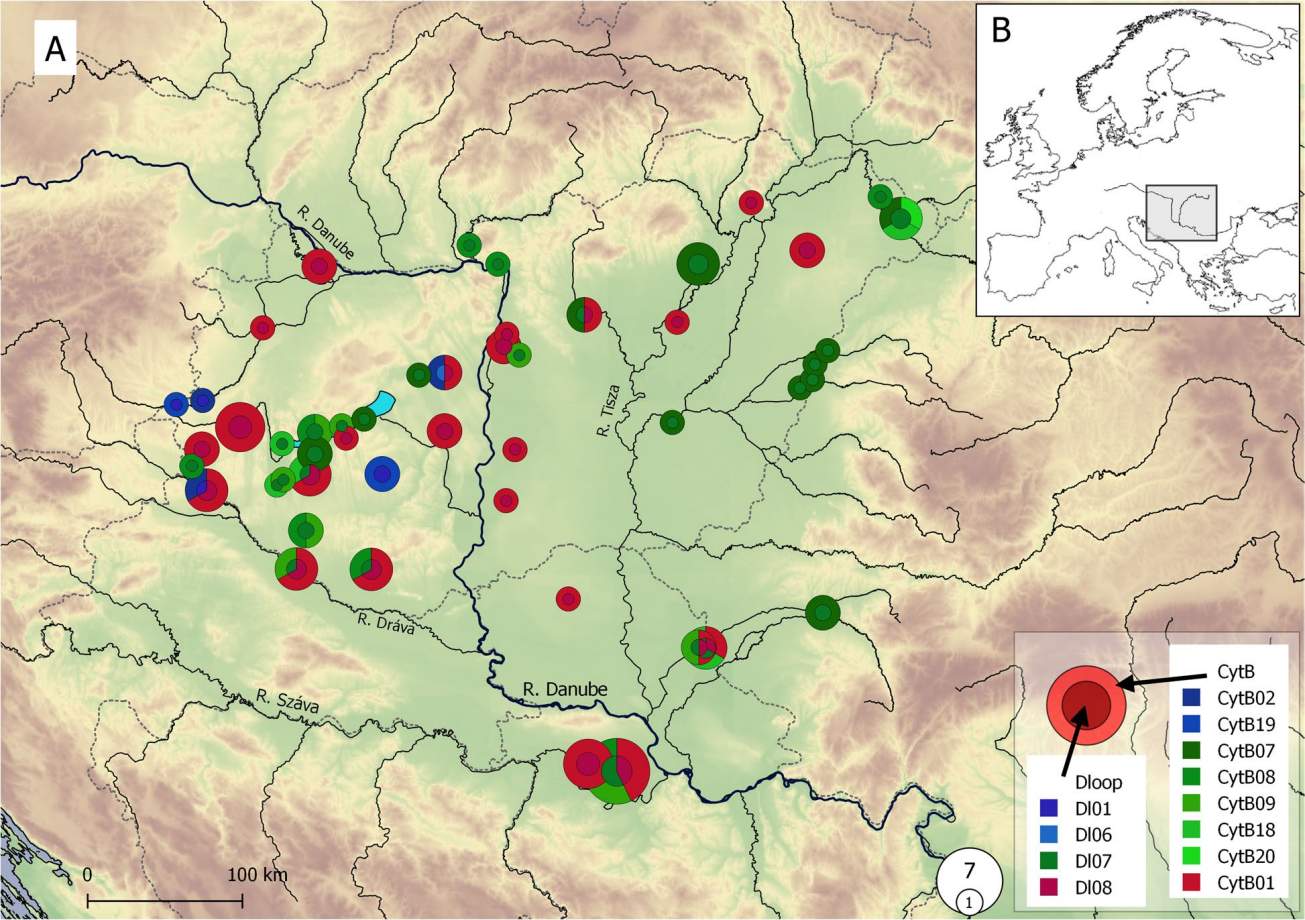
Distribution of the 49 sample sites in the inner area of the Carpathian Basin. (A) and the location of the studied area in Europe (B). The circles’ area corresponds with the number of the sampled individuals. The inner area and the frame of the circles show the D-loop and CytB haplotypes, respectively. The colors and shades of circles indicate their lineage membership (blue shades: Northern, red: Circumpolar, green: Southern). Country borders are shown by grey dotted lines. Base map was generated by QGIS software using layers freely available from European Environment Agency (EEA). Color online only

In the case of D-loop and CytB loci, 436 bp and 1088 bp long sequences were analysed respectively. In these mtDNA sequences altogether 5 and 14 polymorphic (segregating) sites, with no gaps or missing data were indicated. The 88 analysed samples were classified into four (D-loop) and eight (CytB) haplotypes (Table 1, Supplementary table 1). Nucleotide diversity for the entire assemblage ranged from 0.00381 (D-loop) to 0.00418 (CytB). Pairwise genetic differences (uncorrected p-distances) among the indicated haplotypes ranged between 0.0023 and 0.0092, and between 0.0009 and 0.0083 for the D-loop, and CytB loci respectively concerning substitutions (Table 2). Analyses using the MegaBlastN online software [33] showed that all the indicated D-loops, and five out of the eight CytB haplotypes, show a complete match with the ones that can be found in the GenBank database (Table 1). The three newly revealed CytB sequences have been uploaded into GenBank under the accession numbers MZ822418-19–20. The ML trees showed the monophyletic origin of the revealed haplotypes in both cases (Fig. 1), with only slight differences between the D-loop haplotypes. At the same time, the CytB haplotypes were separated into three distinct groups, and findings are reinforced by the results of network analyses (Fig. 2). The individuals showed uneven distribution among the three detected groups (lineages). Only six individuals (6.8%) showed the haplotypes of the Northern lineage while individuals belonging to the Circumpolar and Southern lineage dominated the samples. These two latter lineages are generally distributed in the area. And their abundances differ considerably in the Tisza (Tisa) and Száva (Sava) subdrainages only (Fig. 3). In the former 69% of the individuals, while in the latter 64% of the stock showed Southern and Circumpolar haplotypes respectively. But even in these subdrainages the different lineages may also appear on closely situated sites. Moreover, on nine sample sites stocks with mixed lineage origin were detected (Fig. 3, Supplementary table 1). The Circumpolar and Southern lineages did not show any differences by their geographic distribution patterns and altitude. While the individuals belonging to the Northern lineage appeared from sites situated to more western longitudes (Kruskal–Wallis test, H = 8.752, *p* < 0.01) and from significantly higher altitudes (Kruskal–Wallis test, H = 10.17, *p* < 0.01).

**Table 1:** Clade memberships, codes, and Genbank accession numbers of sequences used for the network analyses. Haplotypes appeared in our samples are italicized. The three newly found CytB haplotypes are marked by asterisk

Lineages	D-loop		CytB	
	**code**	**Genbank acc. no**	**code**	**Genbank acc. no**
**Northern**	*Dl01*	*AJ609603*	*CytB02*	*KM281471*
	Dl02	AJ609604	CytB06	KM281472
	Dl03	AJ609605	CytB17	AY497451
	Dl04	AJ609606	CytB19*	*MZ822419*
	Dl05	AJ609607		
	*Dl06*	*AJ609608*		
**Circumpolar**	*Dl08*	*AJ609610*	*CytB01*	*KM281455*
	Dl12	JX431522	CytB03	KM281462
			CytB04	KM281463
			CytB05	KM281459
			CytB10	KM281458
			CytB11	KM281456
			CytB12	KM281460
			CytB13	KM281461
			CytB14	KM281464
			CytB15	KM281466
			CytB16	KM281465
**Southern**	*Dl07*	*AJ609609*	*CytB07*	*KM281476*
	Dl09	AJ609611	*CytB08*	*KM281478*
	Dl10	AJ609612	*CytB09*	*KM281467*
			CytB18*	*MZ822418*
			CytB20*	*MZ822420*

**Table 2:** Gene diversity and nucleotide diversity for Cytb and D-loop in the entire *E. lucius* assemblage, and in the the three identified lineages. N: number of studied individuals, h: number of haplotypes detected, S: number of segregating sites, Hd: gene diversity ± SD, π and θw nucleotide diversity

Locus	group/variable	N	h	S	Hd ± SD	π ± SD	θw
CytB	Entire assemblage	88	8	14	0.730 ± 0.0390	0.00418 ± 0.00014	0.00255
	European lineage	6	2	1	0.600 ± 0.0167	0.00055 ± 0.00012	0.00040
	Circumpolar lineage	41	1	0	0	0	0
	Southern lineage	41	5	4	0.748 ± 0.0012	0.00123 ± 0.00012	0.00086
D-loop	Entire assemblage	88	4	5	0.569 ± 0.0240	0.00381 ± 0.00010	0.00227
	European lineage	6	2	1	0.333 ± 0.0463	0.00076 ± 0.00050	0.00100
	Circumpolar lineage	41	1	0	0	0	0
	Southern lineage	41	1	0	0	0	0

### Phenotypic features

**Fig. 4:**
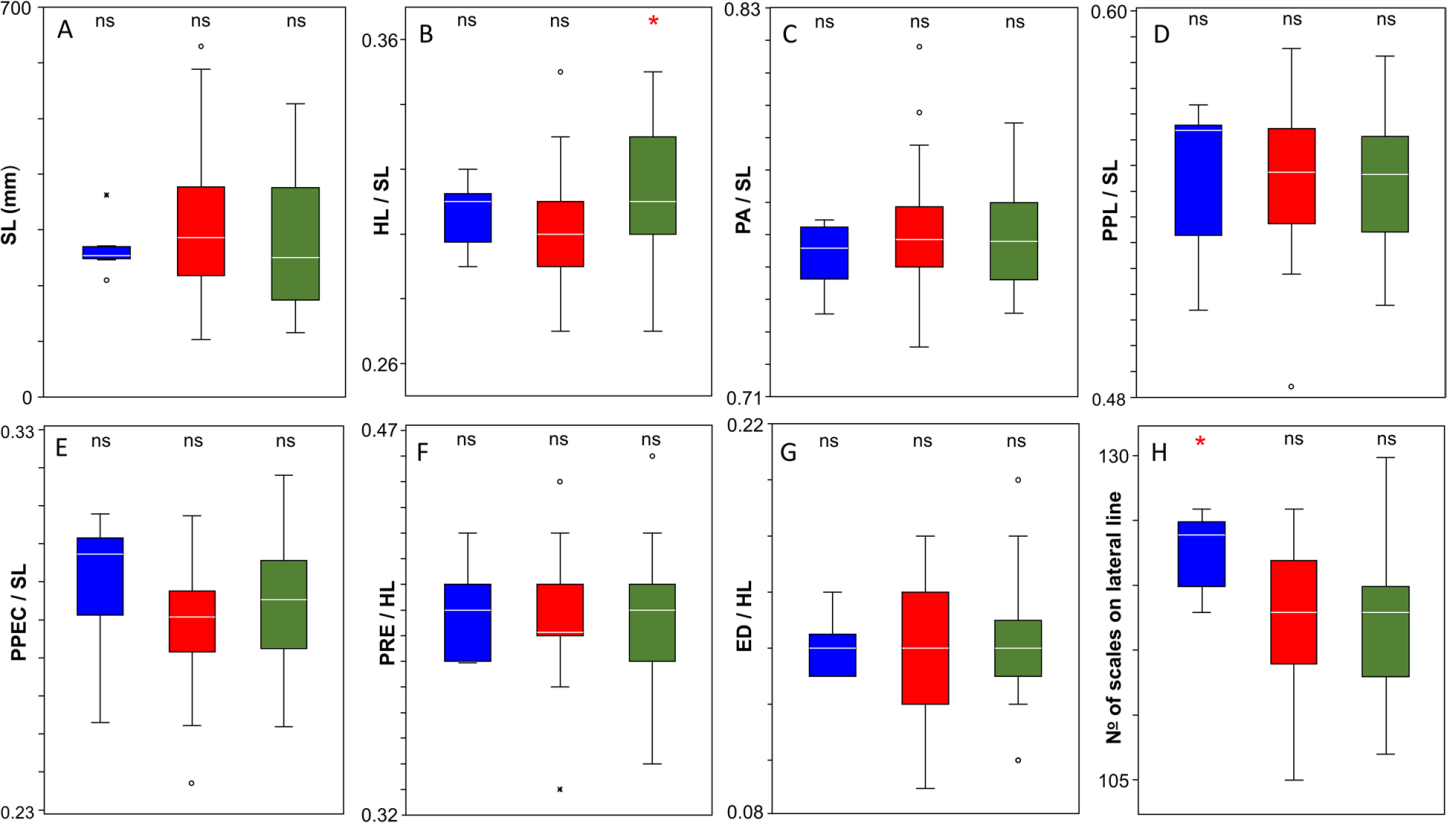
Phenotypic features of the three pike lineages (blue: Northern, red: Circumpolar, green: Southern). SL—Standard lengths (A), HL/SL—Head length in the proportion of Standard Lengths (B), PA / SL—Preanal distances in the proportion of the Standard Length values (C), PPL / SL—Prepelvic distances in the proportion of the Standard Length valuess (D), PPEC / SL—Prepectoral distances in the proportion of Standard Lengths (E), PRE / HL—Preorbital distances in the proportion of the Head Length values (F), ED / HL—Eye diameter in the proportion of the Head Length values (F) and the number of scales on the lateral line (H). Each box represents the 25% and 75% quartiles of the dataset, the band in the box is the median. The whiskers are drawn from the top of the box up to the largest data point less than 1.5 times the box height from the box (the"upper inner fence"), and similarly below the box. Values outside the inner fences are shown as circles values further than three times the box height from the box (the "outer fences") are shown as black stars. Datasets marked by red asterisk differ significantly from the others by the results of non-parametric Kruskal–Wallis pairwise comparisons (*p*˂0.05). Colour figure online

**Fig. 5:**
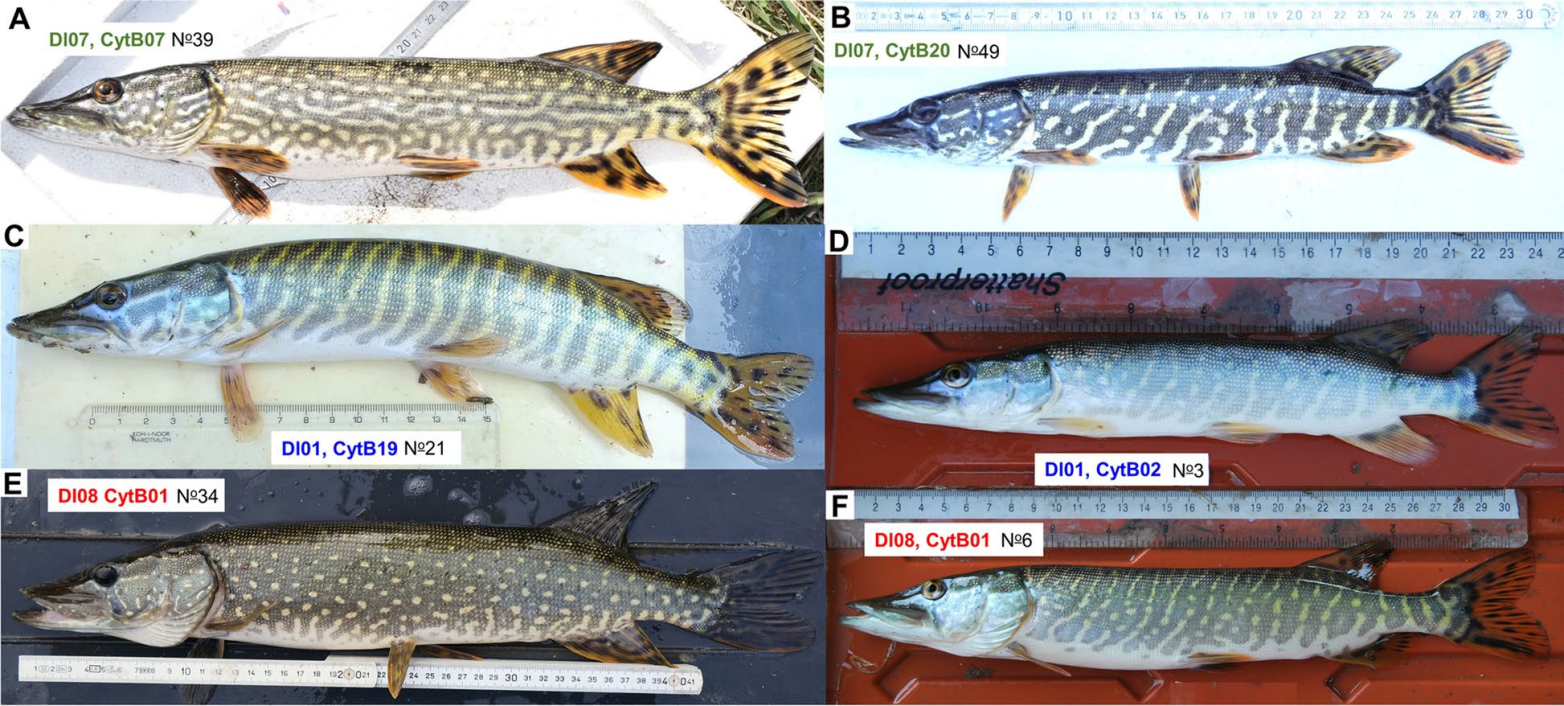
Coloration and body patterns of some representatives of the Southern (A-B), the Northern (C-D), and the Circumpolar (E–F) lineages. Codes beside the presented individuals showing their D-loop and CytB haplotypes, while the N^o^ refer to their collection sites, for more details see Table 1. Color figure online. All photos were taken by the authors

**Fig. 6:**
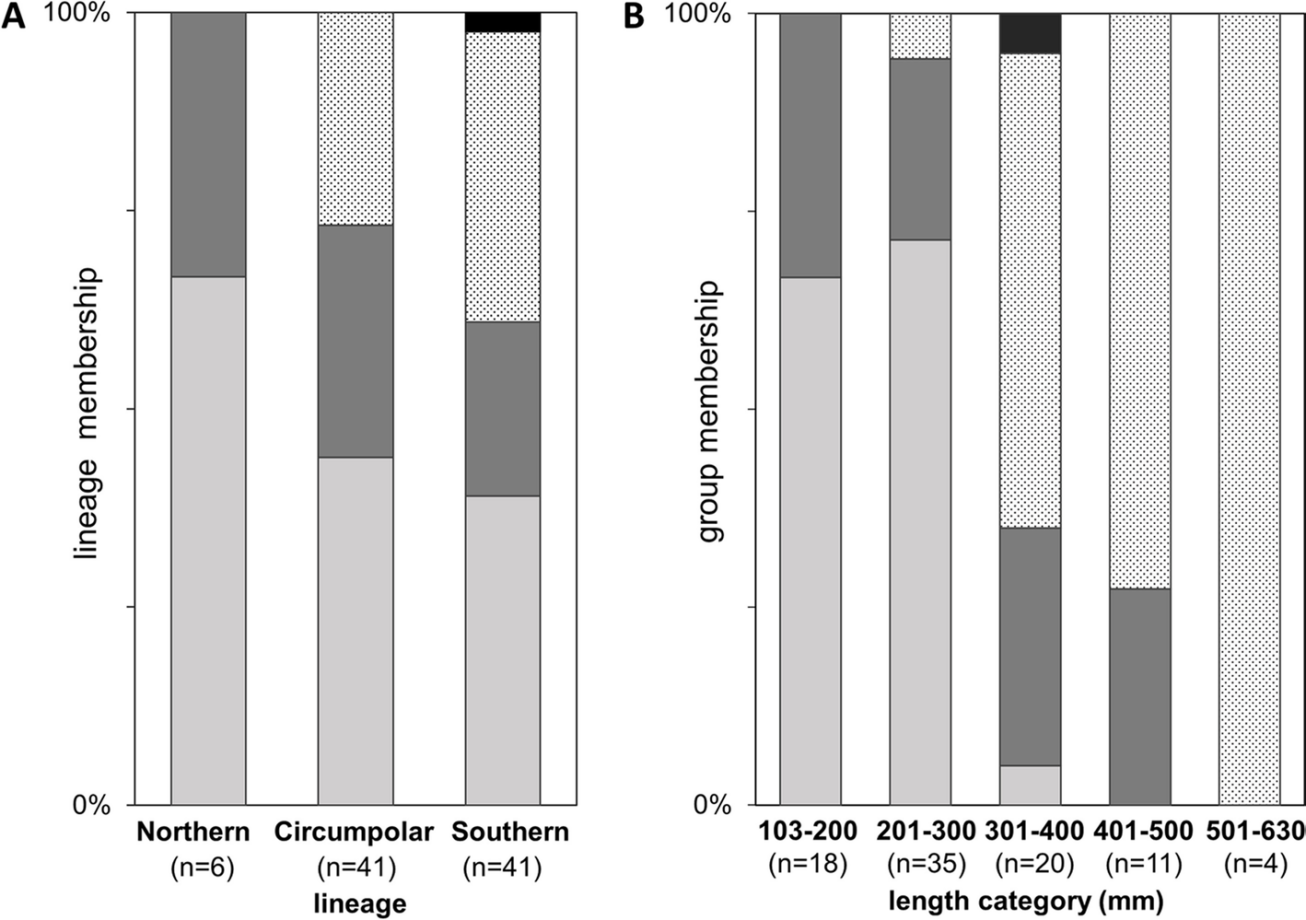
The body pattern features in different phylogenetic clades and lenght group categories. Distribution of individuals characterised by the same body pattern in different phylogenetic clades (A) and in certain size categories (B). Body pattern categories: Light grey: diagonal bars on the body; dark grey: dots arranged to stripes; black dotted: dotted body pattern; black: longitudinal bars on the body

All the investigated phenotypic and morphometric data are available in the Supplementary table 1. The standard body length of the analysed individuals ranged between 103 and 630 mm (mean ± SD: 289.9 ± 110.6 mm). The number of scales on the lateral line ranged between 105 and 130 (mean ± SD: 117.9 ± 5.8). The measured and counted phenotypic features of the three lineages are presented in Fig. 4. In most cases the recorded variables showed high level of within group variations. However, the largest individuals belonged to the circumpolar clade the overlapping SL data showed that no significant differences can be found in bodysize of the three lineages. From the compared phenotypic features only two showed significant differences. The standardized head length (HL/SL) of the Southern lineage and the scale number on the lateral line of the Northern lineage (Kruskal–Wallis test, H = 5.048, *p* < 0.05) showed significantly higher values than that of the other two lineages (Fig. 4.). The body pattern of the sampled individuals showed high level of variation (Fig. 5) but they could be classified into four major groups. The body pattern of 38 individuals showed diagonal bars. In case of 23 pike specimens, the body was dotted but these dots were arranged into stripes. More 26 individuals had dotted body pattern, while only one specimen showed longitudinal bars on its body. The body pattern proportion of the investigated specimens classified into different size groups and clades are shown in Fig. 6.

## Discussion

Notwithstanding that the Carpathian area served as an extra-Mediterranean refugium for numerous species and many endemic species live in this area [34–36], we did not find any unknown pike lineages. All sampled individuals belonged to *E. lucius*, and no other European *Esox* species were identified. At the same time, our results show that all the three known *E. lucius* lineages mentioned by Skog et al. [15] occur in the natural waters of the Carpathian basin (Figs. 1 and 2).

The genetic diversity of the two mitochondrial loci shows considerable differences. For D-loop, only four (two Northern, one Circumpolar, and one Southern) haplotypes were revealed from the study area. Parallelly, eight CytB haplotypes were detected from our samples. This finding might be unusual since the D-loop—although barely half as long as the other sequence analysed—is known to be more variable because it is a noncoding locus [37]. The haplotype diversity of the CytB locus showed variation among the three lineages. The Northern and the Southern lineage had two and five haplotypes, while the Circumpolar clade, in spite of its wide range, had only one haplotype. The haplotype diversity and their specific distribution feature may refer to the origin of the detected lineages. Due to the high genetic variability and the distribution data of the haplotypes of the Southern lineage, it might be the only native lineage in this area.

The other two lineages presumably appeared in the area by natural migration and/or human introduction. The Northern lineage is present mostly in the westernmost area of the Carpathian basin which assumes the recent spread of this lineage eastwards from the Alpin area. At the same time, the fact that the few individuals carrying these haplotypes appeared from distant and isolated places (e.g. Lake Velencei, or Koppány stream) presuppose that anthropogenic effects (introductions) may also play an important role in the river basin spread of this lineage. The fact that this lineage appeared from higher altitudes can be explained by the geographic characteristics of the area (Fig. 3), namely that the westernmost part of the basin is mostly highland.

The Circumpolar lineage is likely to be an introduced one in the Carpathian basin also. This assumption is reinforced by the genetic uniformity of this clade. Moreover, its wide range distribution suggest that this clade might have appeared in the area earlier than the Northern lineage. Considering the international relations of the socialist countries in the field of aquaculture in the mid twentieth century [38, 39], it can be rightly assumed that this lineage introduced from the Soviet union into one or more countries can be found in this area. And these pike stocks exploited the characteristics of the Carpathian water system [40] which allowed the natural spread of this clade in the whole basin. At the same time, the importance of pike in aquaculture and angling, might have facilitated the human-assisted spread of this nonnative lineage also in the study area. Moreover, the stockings are still ongoing in these years also. There are several natural water bodies (e.g. Lake Tisza, Lake Balaton, etc.) in Hungary, where hundreds of thousands of pike juveniles are stocked in every year for angling purposes [41, 42]. Recently we have assumptions only why these, nonnative lineages are stocked in the study area. One possible reason for the human-facilitated spread of the Circumpolar and Northern lineages can be, that they grow larger and/or faster than Southern clade. And these characteristics provide an advantage in the selection of aquaculture breeding lineages [43]. But to test this hypothesis, further studies are required.

The body pattern of the studied individuals showed high variation, but it did not related to clade membership. At the same time it seems to be in correspondence with body size. Namely the Carpathian pike juveniles, similarly to other Northern pike stocks, are mainly striped, and during the ontogeny these transversal stirpes are broken to separate dots, and most of the adults show dotted body pattern [1]. Besides, only two of the studied morphometric traits showed considerable differences among the three lineages. The standardized head length (HL/SL) of the Southern, and the number of scales on the lateral line of the European lineage, showed significantly higher values than the others. While the preorbital distance (PRE/HL), an important discriminating feature of the newly described European pike species [19], and the other studied morphometric variables did not show significant differences among lineages (Fig. 4.). These findings show the indicated three northern pike lineages may be distinguished using certain phenotypic traits, but here we have to emphasize that due to uneven group sizes (6–41-41) and the considerable overlap of these values, these features may only be used if sufficient numbers of individuals are available. Additionally, here we have to note that only mitochondrial markers were used in this study. These are widely used in phylogenetic studies [37, 44–47], although they do not provide any information if hybridisation has taken place between certain lineages. Whereas hybridisation is highly plausible in the study area, since several sampled populations show mixed-lineage origin (see Fig. 3 and Supplementary table 1). The highly variable growth and length–weight relationships of Hungarian pike populations [28, 29] were generally argued to be the consequence of the highly variable environmental conditions of the area. However, knowing these results we can reasonably assume that the detected growth differences may be at least partially due to genetic effects (e.g. the patchy distribution of certain lineages, possible hybridisation). Thus, our results suggest that allopatric pike lineages separated only in the geological near past [15] similarly to other Middle Danubian fish taxa [48, 49], came to secondary contact. This feature provides a potential for rapid adaptation to new environmental conditions [50], at the same time it hinders or reverses the long-term speciation process in its initial steps. In many cases, the introductions through the appearance of new forms and gene variants may even increase the local genetic variability [51]. However, this change may lead to a decrease in infraspecific genetic diversity, so it can be considered as a form of biotic homogenization [52, 53]. Therefore, the human-facilitated introductions of non-native lineages may also contribute to biodiversity loss.

## Conclusions

The geographic distribution and the haplotype diversity data are in correspondence with the suggestion that the Danube drainage could have served as a glacial refugium for the Southern lineage of the northern pike [15]. Although the isolation of the lineages took place in the geological near past only, it has already manifested in some slight phenotypic differences. Additionally, our results suggest that not just the newly described pike species [20, 54] but the slightly isolated northern pike lineages are also threatened by human-facilitated stockings. These human-induced secondary contacts interfere with the million-year-scale speciation processes of evolution. Moreover, results also draw attention to the fact that in the case of economically exploited species the frequency and the spatial resolution of sampling can greatly influence the results of investigations.

## Materials and methods

### Sampling

We collected samples from 49 sites in the Carpathian basin (from Hungary, the Republic of Serbia, and Romania) by electrofishing between 2017 and 2019 (Supplementary table 1, Fig. 1). During our field collections, we paid special attention to the smaller-bodied individuals with unusual body color and pattern. Altogether 88 northern pike individuals’ fin clips were sampled for genetic investigations and stored in 96% ethanol at -20 °C until DNA extraction. After tissue sampling, all specimens were placed flat, and their left sides photographed from a perpendicular angle using a digital camera with a fixed zoom range. All individuals were returned to their original habitats after the sampling procedure.

### Mitochondrial, phylogenetic investigations

DNA was isolated from 10–20 mg of fin tissue per individual using DNeasy Blood and Tissue kit (Qiagen) according to the manufacturer’s instructions. The quality and quantity of the extracted DNA were checked by using a NanoDrop 2000c Spectrophotometer (Thermo Scientific, USA). The mitochondrial D-loop and Cytochrome B (CytB) loci were sequenced for taxonomic and phylogenetic analysis. Amplification of both sequences was performed using Phusion Hot Start II High Fidelity DNA Polymerase (1U) in Phusion Green HF buffer (ThermoFisher Scientific) containing 500–500 nM primers. The primers for D-loop PCR were M13CDL-D (5’ GTAAAACGACGGCCAGTAGCTCCCAAAGCTAAGATTC 3’) and M13PIDL (5’ CAGGAAACAGCTATGACGCTTGGTGGGTAACGAGTC 3’). The PCR protocol was the following: 98 °C for 1 min, then 35 cycles of 98 °C for 10 s, 60 °C for 30 s, 72 °C for 20 s, followed by a final extension at 72 °C for 5 min. Partial CytB region of mitochondrial DNA was amplified using primers LCbEluc14263 (5’ GTCATAATTCTACTCGGACTCTAACC 3’) and RCbEluc15503 (5’ CCTCCAACTTCCGGATTACAAAACCGGCGCTC 3’) [16]. The PCR protocol was 98 °C for 1 min, followed by 35 cycles of 98 °C for 10 s, 63 °C for 30 s and 72 °C for 39 s, and a final extension at 72 °C for 5 min. Following electrophoresis, the PCR product was purified from agarose gel using High Pure PCR Product Purification Kit (Roche) according to the manufacturer’s instruction. Bi-directional sequencing of the amplicons was performed by capillary electrophoresis (ABI 3130 Genetic Analyzer Device, AppliedBiosystems) using the BigDye Terminator v3.1 Cycle Sequencing Kit. The primers used for sequencing were M13F (5’ GTAAAACGACGGCCAG) and M13R (5’ CAGGAAACAGCTATGAC 3’) for D-loop amplicon, while the cytochrome B amplicon was sequenced with primers used for amplification.

Sequences were trimmed manually using FinchTV 1.4.0 (Geospiza) and aligned using MUSCLE [55] as implemented in MEGA X software [56]. Calculation of sequence polymorphism and haplotype detachment was performed using FaBox online software [57]. The obtained sequences were compared with the ones uploaded to GenBank using Blast online software [33]. Gene and nucleotide diversity were computed by DnaSP 6 [58] software. The phylogenetic analyses were made separately for the two loci. To reveal the phylogenetic relationships of our sequences, we analysed them together with already known haplotypes [14, 15, 59, 60]. Therefore, these analyses involved 21 and 13 sequence variants for the CytB and D-loop analyses respectively (Table 2). Initial tree(s) for the heuristic search were obtained automatically by applying Neighbor-Join and BioNJ algorithms to a matrix of pairwise distances estimated using the Maximum Composite Likelihood (MCL) approach based on the Tamura-Nei model [61], and then selecting the topology with superior log likelihood value. The trees are drawn to scale, with branch lengths measured in the number of substitutions per site. All positions containing gaps and missing data were eliminated (complete deletion option). At both loci, ML trees were built using the close relative pike species, *E. reichertii* and *E. cisalpinus* haplotypes (GenBank acc. numbers: KM28145, NC02326, AJ609613, and KR053430) as outgroup sequences. Phylogenetic detachments were tested by 100 bootstrap replications. Sequence divergence was calculated with the maximum composite likelihood method also in MEGA X [56]. Besides median-joining networks were constructed—using 11 (D-loop) and 19 (CytB) haplotypes—in Network 10.2.0.0. software [62]. The possible geographic and altitudinal separation of the detected phylogenetic groups were tested by non-parametric Kruskal–Wallis tests.

### Phenotypic investigations

On the digital images taken from the sampled individuals the standard length (SL), head length (HL), preanal (PA), prepelvic (PPL), prepectoral (PPC) and preorbital distances (PRE) and the eye diameter (ED) were measured, and the number of scales of the lateral line was counted. We chose these variables because these are the most reliable ones in the separation of the European *Esox* species [17–19]. The measurements and counting were carried out in imageJ software [63] for each individual. The individuals were classified into groups by the results of phylogenetic investigations. The measured values were presented in the proportion of SL (HL, PA, PPL, and PPEC) or HL (ED and PRE). The among group differences of the recorded phenotypic variables were tested by non-parametric Kruskal–Wallis tests. The body pattern of the studied individuals was charecterised according to Lucientini et al. [18] and sorted into groups. We used these groups to show the relationships among body pattern, clade memberships, and body size categories.

## Supplementary Information

**Additional file 1:** **Supplementary table 1.** General, geographic, genetic, phenotype and morphometric data of the analysed 88 pike individuals. Name of waterbody and sample site, their coordinates, subdrainage, country of origin, altitude above seal level (Alt. asl). Codes and colors of haplotypes (blue: Northern, red: Circumpolar, green: Southern) correspond with Fig 1-2-3, and Table 1. The collectors’ inicials are identical with the authors’ initials of the ms. Sites which Esox population is charecterised by mixed phylogenetic origin are emphasized with bold letter type.

## Data Availability

All meristic and phenotype data used or analysed during the current study are available in a supplementary file attached to the publication. The newly indicated haplotypes’ sequence data are available in the Genbank on the following accession numbers: MZ822418-19–20. All other analysed sequence data (see: Table 1) are available in the Genbank also.
